# Supplementary material for: A semi-transparent thermoelectric glazing nanogenerator with aluminium doped zinc oxide and copper iodide thin films
Source: Commun Eng. 2024 Oct 15;3:145. doi: 10.1038/s44172-024-00291-4 (PMC11480348; doi:10.1038/s44172-024-00291-4)
Supplement: Supplementary file 2 — Supplementary Information [file 44172_2024_291_MOESM2_ESM.pdf]

A Semi-transparent Thermoelectric Glazing Nanogenerator with Aluminium doped Zinc Oxide and Copper Iodide Thin Films

Mustafa Majid Rashak Al-Fartoos [a], Anurag Roy[a]*, Tapas K. Mallick, Asif Ali Tahir[a]*

[a]Solar Energy Research Group, Environment and Sustainability Institute, University of Exeter, Penryn Campus, Penryn, Cornwall TR10 9FE, U.K.

*Corresponding Authors: a.roy30@exeter.ac.uk (A.R.); a.tahir@exeter.ac.uk (A.A.T.)

Supplementary Information

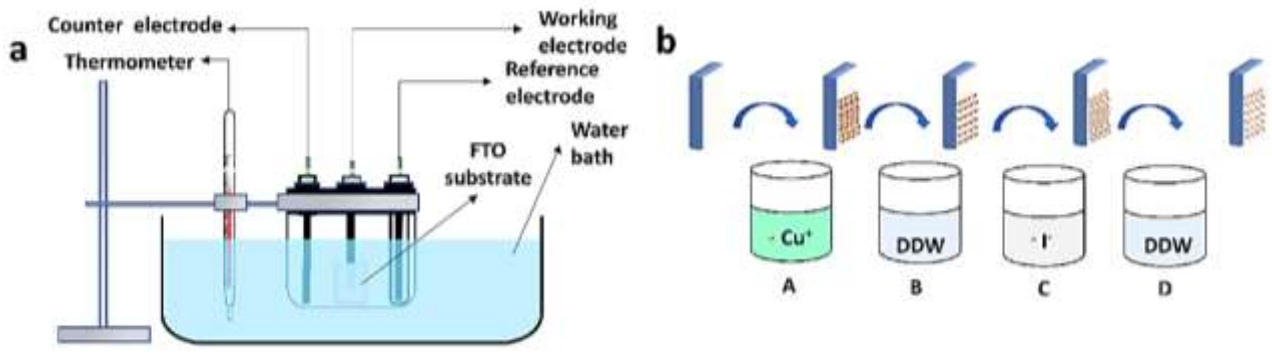

Supplementary Fig 1. a, schematic illustrated the setup of electrochemical depositing of AZO NRs on FTO substrate. **b,** schematic illustrated one SILAR cycle.

Supplementary Table 1. Pure copper, nickel Seebeck coefficient comparison between our homemade device and previously reported values. (Seebeck measurement calibration)				
T (K)	Seebeck (μVK^{-1}) Cu	Seebeck (μVK^{-1}) Cu - this work	Seebeck (μVK^{-1}) Ni	Seebeck (μVK^{-1}) Ni- this work
340	2.2	2.15	-21	-21.5

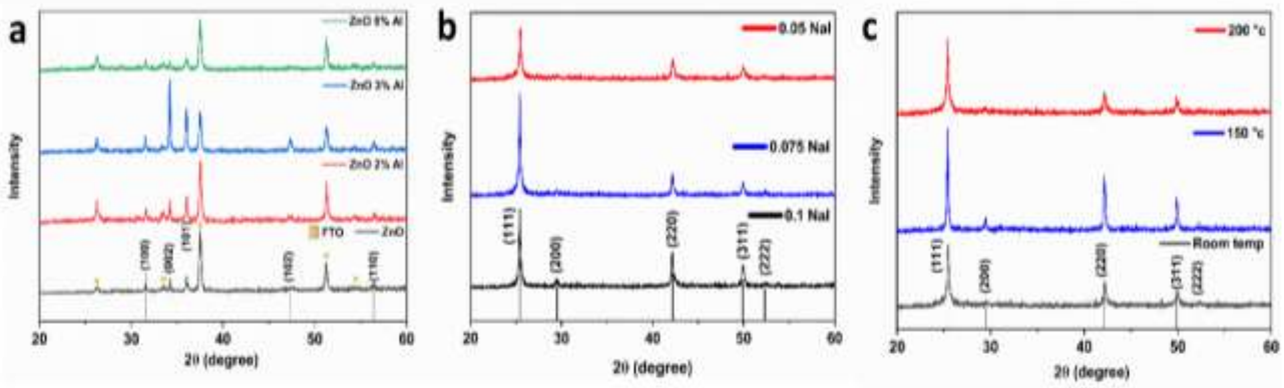

Supplementary Fig. 2. a X-ray diffraction pattern of ZnO samples with different amounts of Al doping as 0, 2%, 3% and 6%. **b** X-ray diffraction pattern of CuI samples where NaI amount varied as 0.05, 0.075 and 0.1 mol. **c** Where the annealing temperature varied as room temperature, 150 °C and 200 °C.

Supplementary Table 2. The Crystallite size extracted from XRD patterns of ZnO thin films with different Al doping ratios.			
Sample	Crystallite size (nm)	a (Å)	c (Å)
ZnO	36.66	3.2713	5.16
AZO 2%	36.34	3.2712	5.21
AZO 3%	31.51	3.2687	5.23
AZO 6%	22.363	3.2667	5.24

Supplementary Table 3. The crystallite size was extracted from XRD patterns of CuI thin films with different NaI ratios and annealing temperatures.		
Sample	Crystallite size (nm)	a (Å)
0.05 Na	23.0	6.0641
0.075 Na	25.7	6.0547
0.1 Na	28.6	6.0643
Annealing at 200	38.4	6.1015
Annealing at 150	31.2	6.0627
Annealing at room temp	25.7	6.0547

Supplementary Table 4. Illustrate the thin film thickness of different samples.	
Sample	Film thickness
AZO 3% 0.5 h	470 nm
AZO 3% 1h	700 nm
AZO 3% 1.5 h	800 nm
CuI 30 cycle	1.7 µm
CuI 35 cycle	1.96 µm
CuI 40 cycle	2.68 µm

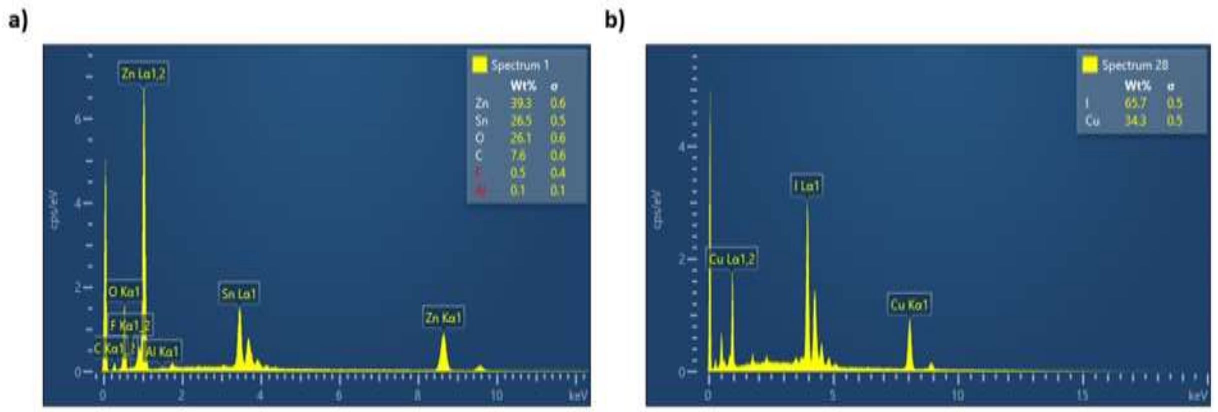

Supplementary Fig. 3. X-ray energy-dispersive spectrum of **a** AZO 3% and **b** CuI at 0.075 (M) NaI thin films, respectively.

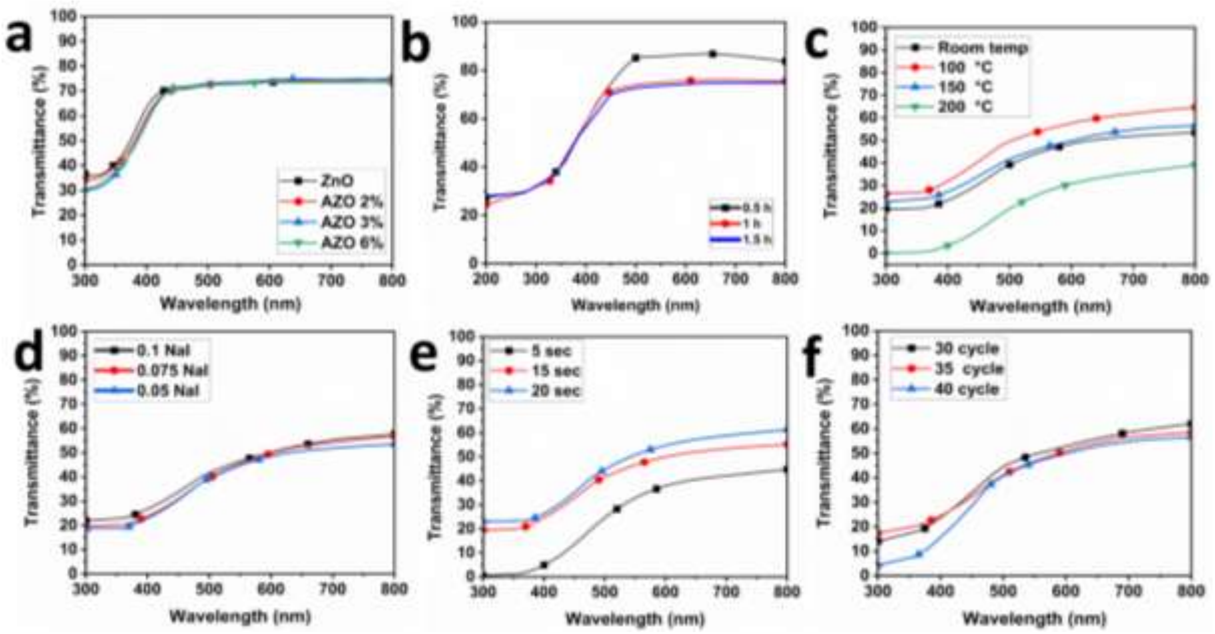

Supplementary Fig. 4. The transmission spectrum of the AZO thin film is plotted against different Al concentrations. **b** the graph depicts the AZO thin film's transmission as influenced by different deposition durations. The thickness factor plays a role in determining the thin film's transparency. **c** Film's transmission spectrum at varying annealing temperatures. **d** The influence of different NaI concentrations on the transmission of CuI thin films. **e** The transmission spectrum of the CuI thin film against various dipping times. **f** The effect of different cycling durations on the CuI thin film's transmission spectrum.

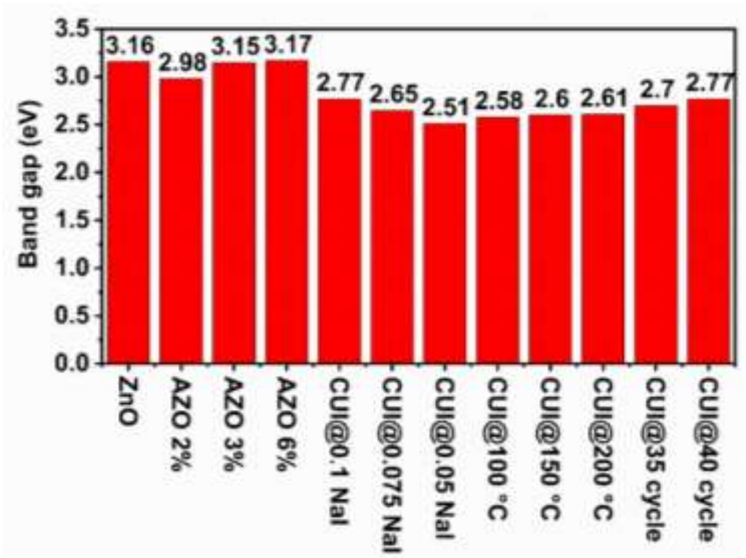

Supplementary Fig. 5. A comparative graph compares the band gaps of various AZO and CuI thin film samples, facilitating a holistic understanding of how different compositions and treatments modify electronic properties.

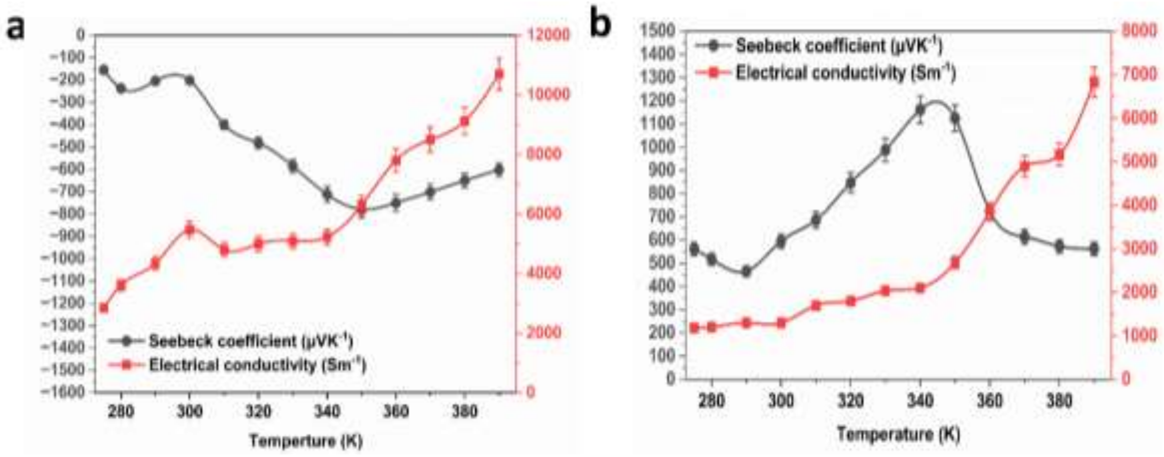

Supplementary Fig. 6. Temperature dependence of the Seebeck coefficient and electrical conductivity measurements a, AZO and **b,** CuI thin films, respectively.

Durability of thermoelectric glazing nanogenerator

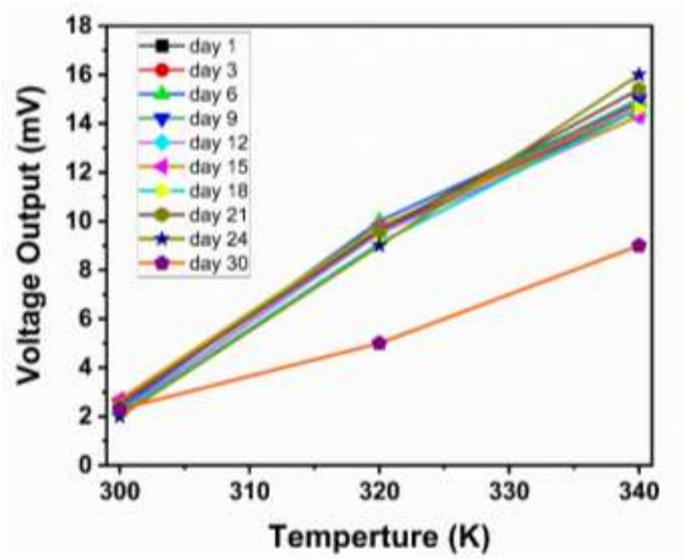

Supplementary Fig. 7. the output voltage of AZO-CuI thermoelectric nanogenerator up to 30 days.
