# Supplementary material for: A semi-transparent thermoelectric glazing nanogenerator with aluminium doped zinc oxide and copper iodide thin films
Source: Commun Eng. 2024 Oct 15;3:145. doi: 10.1038/s44172-024-00291-4 (PMC11480348; doi:10.1038/s44172-024-00291-4)
Supplement: Supplementary file 3 — Description of Additional Supplementary Files [file 44172_2024_291_MOESM3_ESM.pdf]

Description of Additional Supplementary Files

File name: Supplementary Data 1
Description: All data used to create the figures in the main article and supplementary information.
